# ^1^H, ^13^C, and ^15^N assignments of the mRNA binding protein hnRNP A18

**DOI:** 10.1007/s12104-022-10117-z

**Published:** 2022-12-21

**Authors:** Katherine M. Coburn, Braden Roth, Kristen M. Varney, France Carrier, David J. Weber

**Affiliations:** 1 Department of Biochemistry and Molecular Biology, University of Maryland School of Medicine, 108 N. Greene Street, 21201 Baltimore, MD USA; 2 Department of Radiation Oncology, University of Maryland School of Medicine, 655 West Baltimore, Street, 21201 Baltimore, MD USA; 3 University of Maryland Marlene and Stewart Greenebaum Comprehensive Cancer Center, 21201 Baltimore, MD USA; 4 Center for Biomolecular Therapeutics (CBT), Department of Biochemistry and Molecular Biology, University of Maryland School of Medicine, 108 N. Greene St, 21201 Baltimore Maryland, USA

**Keywords:** RBP (RNA binding protein), hnRNP A18 (heterogeneous ribonucleoprotein A18), CIRBP (cold inducible RNA binding protein), IDD (intrinsically disordered domain)

## Abstract

Heterogeneous ribonuclear protein A18 (hnRNP A18) is an RNA binding protein (RBP) involved in the hypoxic cellular stress response and regulation of cytotoxic T-lymphocyte-associated protein 4 (CTLA-4) expression in melanoma, breast cancer, prostate cancer, and colon cancer solid tumors. hnRNP A18 is comprised of an N-terminal structured RNA recognition motif (RMM) and a C-terminal intrinsically disordered domain (IDD). Upon cellar stressors, such as UV and hypoxia, hnRNP A18 is phosphorylated by casein kinase 2 (CK2) and glycogen synthase kinase 3β (GSK-3β). After phosphorylation, hnRNP A18 translocates from the nucleus to the cytosol where it interacts with pro-survival mRNA transcripts for proteins such as hypoxia inducible factor 1α and CTLA-4. Both the hypoxic cellular response and modulation of immune checkpoints by cancer cells promote chemoradiation resistance and metastasis. In this study, the ^1^ H, ^13^ C, and ^15^ N backbone and sidechain resonances of the 172 amino acid hnRNP A18 were assigned sequence-specifically and provide a framework for future NMR-based drug discovery studies toward targeting hnRNP A18. These data will also enable the investigation of the dynamic structural changes within the IDD of hnRNP A18 upon phosphorylation by CK2 and GSK-3β to provide critical insight into the structure and function of IDDs.

## Biological Context

Heterogeneous ribonuclear protein A18 (hnRNP A18), also known as cold inducible RNA binding protein (CIRBP) is an RNA binding protein (RBP) differentially upregulated in breast, melanoma, pancreatic, and colon solid tumors in response to low oxygen tension (Chang et al., 2016; Pamboukian, 2011; Yang and Carrier, 2001; Yang et al., 2006; Yang et al., 2010). In response to cellular stress, such as UV or hypoxia, hnRNP A18 translocates from the nucleus to the cytosol where it stabilizes target mRNAs for pro-survival genes, such as hypoxia inducible factor 1-α (HIF1-α), thioredoxin (TRX), and cytotoxic T-lymphocyte-associated protein 4 (CTLA-4) (Chang et al., 2016; Solano-Gonzalez et al., 2021). HIF-1α is the master regulator of the hypoxic cellular response, which controls gene expression for functions such as angiogenesis, tumor metastasis, cellular metabolism, glucose uptake, cellular proliferation, cellular differentiation, and apoptosis (Rankin and Giaccia, 2016; Rankin et al., 2016; Semenza, 2012). Elevated expression of HIF-1-α and hnRNP A18 is associated with poorer cancer patient prognosis (Chang et al., 2016; Rankin and Giaccia, 2016). Applications that either directly or indirectly decrease HIF1-α expression and are utilized in combination with other anti-cancer therapies have demonstrated an increase in response to radiotherapy and chemotherapy (Tang and Zhao, 2020). However, there are currently no FDA approved therapies that target HIF-1-α. CTLA-4 is an immune checkpoint receptor that downregulates the cellular immune response toward self-tissues and treatment of patients with anti-CTLA-4 antibodies, such as Ipilimumab, have demonstrated increased progression-free survival in late stage metastatic melanoma, when compared to traditional chemotherapeutics alone (Lipson and Drake, 2011; Walunas et al., 1994). To address the unmet need of HIF-1-α inhibition, but also combine the therapeutic benefits of immunotherapy modulation, potent and selective hnRNP A18 inhibitors are needed to provide effective treatment options for patients with disease refractory to immunotherapy modulators.

hnRNP A18 is an 18.6 kDa protein that contains an RNA binding domain (RBD), consisting of an RNA recognition motif (RRM) (aa. 1–89), and an intrinsically disordered domain (IDD) (aa. 90–172). Upon cellular stressors, casein kinase 2 (CK2) and glycogen synthase kinase 3β (GSK-3β) phosphorylate hnRNP A18 in the nucleus. hnRNP A18 translocates from the nucleus to the cytosol where it interacts with target mRNAs (Yang et al., 2006). The RRM of hnRNP A18 recognizes target mRNAs with a 52 nucleotide hnRNP A18 consensus sequence and interacts with the nitrogenous bases of such targets through conserved aromatic residues within two ribonucleoprotein consensus sequences (Creigh-Pulatmen 2014; Yang et al., 2006; Yang et al., 2010). However, the strongest interactions between hnRNP A18 and target RNAs require both the RRM and IDD (Yang et al., 2010). Recently, a multi-disciplinary team developed hnRNP A18 specific small molecule inhibitors that disrupt hnRNP A18 from interacting with target mRNAs (Solano-Gonzalez et al., 2021). These inhibitors were identified through the computer aided drug design (CADD) through a site identified ligand competitive saturation pharmacophore (SILCS-Pharm) protocol, which developed pharmacophore models that exploit the X-ray crystal structure of the hnRNP A18 RRM (aa. 1–91) (Coburn et al., 2017; Guvench and MacKerell, 2009; Raman et al., 2011; Raman et al., 2013; Yu et al., 2015). Over 720,000 potential drug-like compounds were screened against the pharmacophore models and 264 compounds were identified for further investigation based on their chemical and physical properties. NMR investigations produced lead compounds that subsequently demonstrated specificity and inhibition of hnRNP A18 (Solano-Gonzalez et al., 2021).

However, structural, and dynamic changes of hnRNP A18 in the presence of posttranslational modifications (PTMs), such as phosphorylation by CK2 and GSK-3β, may impact small inhibitor binding dynamics. The sequence-specific backbone and sidechain resonance assignments for hnRNP A18 were completed as a step toward probing dynamic changes in the structure and function of hnRNP A18 upon phosphorylation by CK2 and GSK-3β. These data are important for the longer-term goal of designing higher affinity and more selective small molecule inhibitors for hnRNP A18 that will enable both targeting of the hypoxic cellular response and the immune modulatory pathways exploited by cancer cells.

## Methods and experiments

### Protein expression and purification

hnRNP A18 was cloned into the *Escherichia coli (E. coli)* expression plasmid pET21a in frame with a 6x-His tag upstream. The pet21a-His_6_hnRNPA18 construct was transformed into E. coli BL21(DE3) cells and a single colony was grown in 5 L of M9 minimal medium (Sambrook and Russell 2006) at 37 ºC with ^15^ N-labeled (> 99%) ammonium chloride (0.5 g/L) as the single nitrogen source and ^13^ C-labeled (> 99%) D-glucose (2.0 g/L) as the single carbon source. When the A_600_ reached 0.8, the incubation temperature was reduced to 18 ºC. His_6_-hnRNP A18 expression was induced by the addition of 1 mM IPTG (isopropyl-β-D-1-thiogalactopyranoside), and cells were grown for an additional 16 h. Cells were pelleted by centrifugation at 10,000 x g for 20 min and resuspended in lysis buffer (20 mM Tris pH 7.4, 0.5 M NaCl, 5 mM Imidazole, 6 M urea and 1 mM PMSF). The resuspended cells were sonicated and subsequently centrifuged at 18,000 x g for 45 min to pellet cellular debris and the supernatant was filtered with a 0.45 μm syringe. Protein purification was achieved through Ni-affinity chromatography. A hand poured 10 mL Ni Sepharose 6 Fast Flow (GE Healthcare, catalog number 17-5318-01) column was equilibrated with lysis buffer and loaded with the filtered cleared lysate. The column was washed with 10 volumes of 20 mM Tris pH 7.4, 0.5 M NaCl, 2.5 M Urea and 30 mM Imidazole to remove non-specific protein interactions. His_6_-hnRNP A18 was eluted from the column with 10 volumes of 20 mM Tris pH 7.4, 0.5 M NaCl, 2.5 M urea, and 250 mM Imidazole. Relevant fractions were combined and His_6_-hnRNP A18 was refolded by dialysis against 2 × 4 L of 50 mM acetic acid pH 5.2 for 4 h each at room temperature. The dialyzed supernatant was filtered through a 0.2 mm syringe and concentrated via a 10 kDa MWCO centrifugal concentrator (Amicon Ultra-15 10 K, catalog number 516–0556).

### NMR spectroscopy

Standard Bruker pulse sequences for HNCA, HN(CO)CA, HNCO, HN(CA)CO, HNCACB, CBCA(CO)NH, HCCH_TOCSY, HC(CO)NH, C(CO)NH, and ^15^ N-HSQC experiments were performed on either a Bruker Avance III 950 MHz or a Bruker Avance III 600 MHz spectrometer, each equipped with z-gradient TCI cryogenic probes. All experiments were performed in 50 mM acetic acid pH 5.2 at 298 K and 10% D_2_O was added to the sample prior to collection of the triple resonance experiments. All proton chemical shift values were referenced to external trimethylsilyl propanoic acid at 25 °C (0.00 ppm) with respect to residual H_2_O (4.698 ppm). All standard 3D assignment experiments were processed using NMRPipe (Delaglio et al., 1995) and analyzed by CCPNmr (Vranken et al., 2005). Talos-N was used to determine secondary structure probabilities based on experimentally derived HN, N, Cα, Cβ and C′ contours (Shen and Bax, 2013).

### Extent of assignment and data deposition

**Fig. 1:**
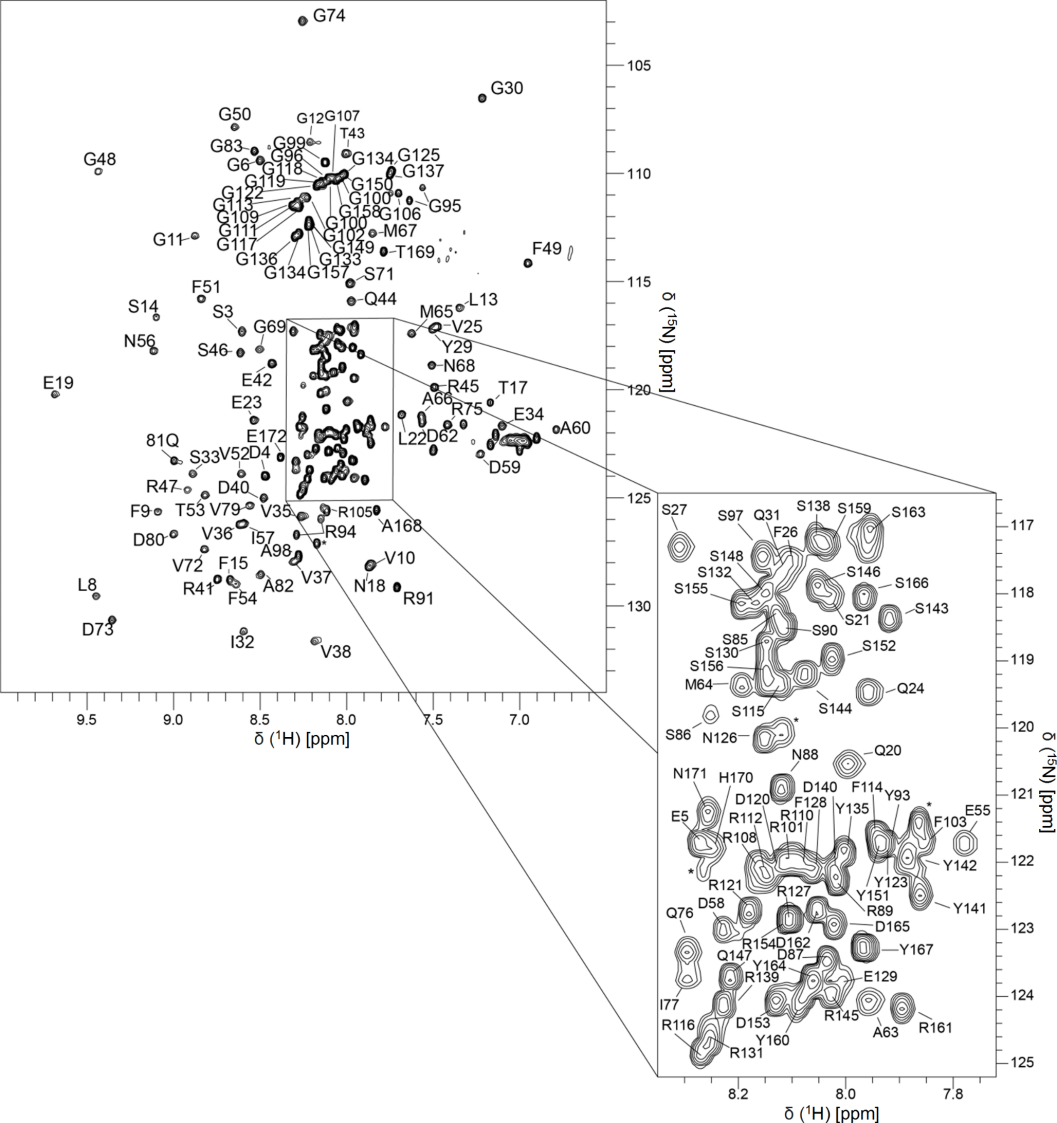
**Resonance assignments of hnRNP A18.** The 2D ^1^ H,^15^ N-edited HSQC spectrum of hnRNP A18 (residues 1-172) was recorded on a Bruker 600 MHz spectrometer at pH 5.2 and 25 ^o^C. Residue type and number indicate assignments from the backbone amide H^N^ correlations. Correlations arising from non-native N-terminal histidine residues are labeled with an asterisk (*)

Sequence-specific resonance assignments shown in Fig. 1 were determined unambiguously using heteronuclear multidimensional NMR methods for 160 out of 172 possible H^N^-^15^ N correlations (~ 93%) of hnRNP A18. Of those 160 correlations, 93% of the Cα, 88% of the Cβ, and 91% of C′ chemical shifts were determined. It was also possible to assign 4 of the 6 residues in the N-terminal His-tag, which are labeled with an asterisk (*) in Fig. 1. Five of the 12 residues that do not appear in the 2D ^1^ H-^15^ N-edited HSQC spectrum are either in a short unstructured region of the hnRNP A18 N-terminus (Met1, Ala2), within loops between the α-helices and β-strands of the hnRNP A18 RRM (Asp16, Arg 78), or are located within the IDD (Gly92 and Phe104). Six of the 12 residues not observed in the 2D ^1^ H-^15^ N-edited HSQC spectrum are lysine residues (Lys7, Lys28, Lys 39, Lys 61, Lys70, and Lys 84). It is likely that these missing correlations were the result of conformational averaging occurring on the chemical shift time scale. Two residues (Gly95 and Arg94) at the beginning of the IDD in the RGG motif, an arginine and glycine rich sequence, were each found to have two H^N^ correlations having different ^1^ H and ^15^ N chemical shift values with varying intensities (~ 2:1), but the chemical shift values for their respective pairs of inter- and intra-residue carbon correlations to carbon (i.e. HNCA, HNCACB, etc.) were identical, which suggests that there are potentially two slightly different backbone chemical environments for these two residues. The doubled chemical shifts for each respective residue are within only a few tenths of a ppm. However, providing data for a foolproof conclusion to the doubling is beyond the scope of this assignment note and requires additional experimentation that will be reported elsewhere.

**Fig. 2:**
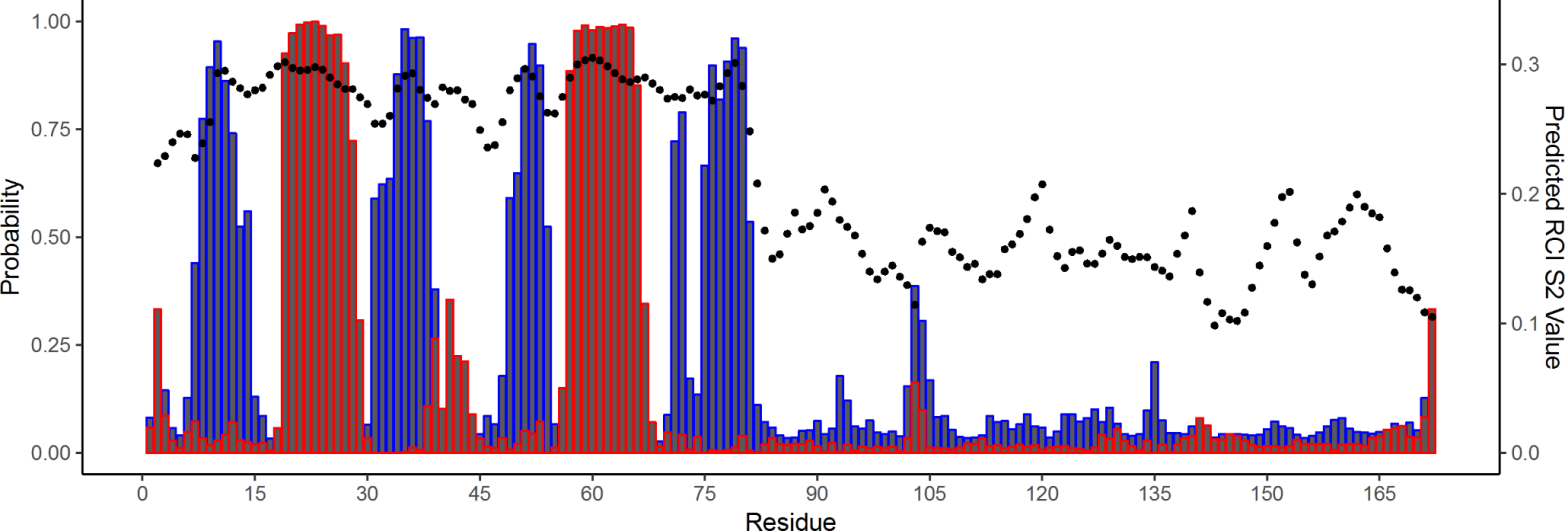
**The probability of secondary structure formation as predicted by Talos-N.** α-helical character is represented by red and β-strand by blue. The random coil index is represented by black circles

In order to further investigate the secondary structural elements of hnRNP A18, the chemical shift assignments of backbone atoms (HN, Hα, Cα, Cβ, CO, and N) for each assigned residue in the sequence were analyzed with TALOS + software (Shen and Bax, 2013) in Fig. 2. The secondary structural elements determined by NMR for the RRM (aa. 1–89) are consistent with the X-ray crystal structure, which demonstrated two α-helices and four antiparallel β-strands with a *β*_*1*_*α*_*1*_*β*_*2*_*β*_*3*_*α*_*2*_*β*_*4*_ alignment (Coburn et al., 2017). Such secondary structure for hnRNP A18 and is also similar to RRMs found within other RBPs (Maris et al., 2005). The IDD of hnRNP A18 (aa. 90–172) contained chemical shift values consistent with random coil and did not suggest strong secondary structural characteristics at any location. In accordance with chemical shifts suggestive of random coil, the Random Coil Index (RCI) order parameter (RCI-S^2^) values for the IDD were lower than the values for the RRM. This analyses suggests the backbone of the IDD is significantly more flexible than the backbone of the RRM.

In summary, the chemical shift values for backbone and sidechain resonances of hnRNP A18 obtained here were deposited in the Biological Magnetic Resonance Bank database (http://www.bmrb.wisc.edu) under accession number 51,517.These data will be important for NMR studies that investigate the structure and function of hnRNP A18 upon post-translational modifications, such as phosphorylation by CK2 and GSK-3β, and design of hnRNP A18 specific small molecule inhibitors.

## Data Availability

NMR chemical shift data are available at the Biological Magnetic Resonance Bank database (http://www.bmrb.wisc.edu) under accession number: 51,517.

## Code Availability

No applicable.
